# Hospital Meetings, &c.

**Published:** 1900-05-19

**Authors:** 


### HOSPITAL MEETINGS, &C.

### VICTORIA HOSPITAL FOR CHILDREN.

Earl Cadogan presided at Grosvenor House on Monday, 14th inst., at the thirty-third annual meeting of the Victoria Hospital for Children. In opening the proceedings he referred to the death of the late Duke of Westminster, and reminded the meeting of the interest he had always taken in that institution. It was to be feared that hospitals generally were suffering from the diversion of subscriptions to the various funds connected with the war. This was not a creditable state of public feeling, since, though many were giving to the full extent of their power, there were many others who could at the same time continue their customary subscriptions to charitable institutions. Otherwise, if contributions were merely transferred from the hospitals to the war funds, it was really the older charities that were assisting these funds. For many years it had been felt that the Victoria Hospital was not suitable for its purpose, and the governors had reluctantly come to the conclusion that it was absolutely necessary to rebuild it so as to put it in a condition to carry on its work in an adequate and proper manner. It was only about five months ago since, in that room, his late friend the Duke of Westminster had presided at a meeting at which all the details of the proposed scheme were explained, and the necessity for the steps they were taking. Then it had been arranged that a dinner should be held, in November last, to advocate the claims of the institution, at which Lord Roberts was to preside. But it so happened that at the date fixed it was quite clear that the interest of the public was concentrated on matters connected with the war, and it was considered advisable to postpone it. Since then Lord Roberts had been absent from England, so that opportunity had been lost. Now it was thought that they should not longer delay laying their case before the public. They felt it should be made clear that in the south-west district the Victoria Hospital was now the only one that could undertake the work of a special hospital for the treatment of children. They found themselves with an old, dilapidated building which required renewal. They were in the midst of a large and increasing population, and they had lost by removal another hospital which formerly did some of the work they now had to do. Altogether, they were in a position which was an exceedingly strong one for an urgent appeal to the public. He called on Mr. Martin Smith, the chairman of the committee of management, to move the report and accounts.

Mr. Martin Smith said one of the first and most important items in the report had reference to the frequent and mysterious outbreaks of diphtheria that had taken place. He was glad to say that the precautions they had taken had stopped these outbreaks, and the health and sanitation of the hospital were now all that could be wished for. But this result had only been attained by the reduction of the number of beds from 60 to 42. This was no doubt a wise, and possibly a necessary step, but inasmuch as the 60 beds they formerly had were insufficient to meet the wants of the large district they served, it was clear that the 42 they now had were wholly insufficient. And it was most unfortunate that the reduction took place when they were threatened with the removal of the Belgrave Hospital. The necessity for this reduction had shown them the necessity for carrying out their proposed plans at the earliest possible moment. Their present building was never intended for a hospital, and was not only wholly unfitted for the work but afforded quite inadequate accommodation. Their out-patients last year numbered 17,000, and to provide a proportionate number of beds they would require at least three times the number they now possessed. The estimated cost of the new building had greatly increased owing to the rise in prices in the building trade, and they had been compelled to submit to modifications of their plans. Even now the cost would come out at from £23,000 to £26,000. To meet this they had altogether between £11,000 and £12,000, leaving some £16,000 to complete. This was a heavy task, and the committee required the warm and hearty co-operation of all the governors. Their object was to succour little children at the most critical period of their lives, at the point which would decide whether they would be sound and healthy members of society, or cripples and invalids—a burden to others and a misery to themselves. In that south-western district there were thousands of men and women who owed all their happiness to the help they had in their young days received at that hospital. He was quite sure that one visit to their wards would change the most apathetic and indifferent among them to an earnest supporter. Theirs was not a new and untried hospital. This was their thirty-third report; upwards of 31,000 children had occupied beds in their wards, while their out-patients had numbered over a million. Their work in the past was well known, but they had now arrived at a point when they could not pretend to cope with it without fresh appliances and a largely-increased number of beds. He would never believe that the wealthy inhabitants of the south-western district—the richest quarter of London—would allow it to remain without adequate provision for the needs of the sick and suffering children of the poor around them. Of course, they were appealing at an unfortunate time, but in spite of the generous response that had been made on behalf of our soldiers and sailors he felt sure there was ample left for their comparatively trifling wants. Turning to the remainder of the report, the committee had great pleasure in expressing to Miss Watson, the lady superintendent, who had succeeded Miss Hamilton on her appointment as matron to University College Hospital, their gratification at the excellent services she had rendered, and also for that of their very efficient staff of nurses. Ill-health had compelled the resignation of Mr. Cameron Skinner, their secretary, and they had sustained great loss in the death of the Duke of Westminster and Viscount Clifden. The accounts showed that the ordinary income for the past year amounted to £4,543, the extraordinary receipts being £1,552. The ordinary expenditure was £4,881, and the extraordinary expenditure £1,403. Of this, £544 was spent on the sanitary improvements already mentioned.

Lord Carrington seconded the motion, which was carried; and a vote of thanks to the chairman, proposed by Lord Sidmouth, terminated the proceedings.

